# Measuring the Effect of Blockchain Extrinsic Cues on Consumers’ Perceived Flavor and Healthiness: A Cross-Country Analysis

**DOI:** 10.3390/foods10061413

**Published:** 2021-06-18

**Authors:** Marco Francesco Mazzù, Veronica Marozzo, Angelo Baccelloni, Flaminia de’ Pompeis

**Affiliations:** 1 Department of Business and Management, LUISS Guido Carli University, Viale Romania, 32-00197 Rome, Italy; abaccelloni@luiss.it (A.B.); fdepompeis@luiss.it (F.d.P.); 2 Department of Economics, Università degli Studi di Messina, Piazza Pugliatti, 1-98122 Messina, Italy; veronica.marozzo@unime.it

**Keywords:** food, flavor, healthiness, blockchain, tastiness, consumer acceptability, product cues, packaging

## Abstract

Many studies in the related literature have proven that the perception of flavor and healthiness can be affected by both the product’s intrinsic and extrinsic cues. Package designs, brands, colors, labels and other visual elements exert and influence consumers’ expectations and guide them toward food decisions. With the increasing initiatives promoted within Europe in support of the adoption of blockchain technology in supply chains and agri-food contexts, in the coming years, packages will be used with additional product information certified with the technology itself. Cueing packages with blockchain-certified information could affect consumers in their overall flavor and health perceptions, similarly to that previously demonstrated with other extrinsic cues. In the present study, we analyzed a sample of 310 primary grocery shoppers from Germany, Italy and the UK, demonstrating the effectiveness of technology-certified information on the package of animal milk in influencing consumers’ flavor and health perceptions and exploring the differences and similarities across the three countries and milk categories.

## 1. Introduction

Technology is progressively intensifying its presence and relevance in several fields, including the food industry, ranging from increasing productivity to improving product quality and supply chain traceability [1]. A relevant area, with limited coverage in terms of past academic literature, is related to how the use of technology, and its associated cues, might influence consumers’ decision-making, with specific attention to the impact on flavor and health perception.

Nowadays, there is an ongoing debate about what could be considered healthy, both in absolute terms and as derived from external signals provided to customers. According to Plasek et al. (2020) [2], several factors might influence such perceptions: (a) communicated information; (b) shape and color of the product packaging; (c) ingredients of the product; (d) product category; (e) organic origin of the product; and (d) the taste and other sensory features of the product.

Furthermore, several authors discussed the relationship between food perceived healthiness and taste, highlighting the mutual effect between the two constructs [3,4]. Specifically, previous findings exhibited a negative relationship between healthiness and taste, supporting the common lay belief named “the Unhealthy = Tasty intuition” (UTI): certain consumers believe that less healthy products taste better and are more enjoyable during consumption [4]; while other studies demonstrated that consumers rate some healthier foods as tastier, consequently showing the existence of a positive relationship between healthiness and tastiness, in opposition to UTI [5,6,7]. However, different tastiness and healthiness perceptions occur according to stimuli—from both internal and external sources—decoded by consumers when evaluating, purchasing and consuming foods [3].

Generally, the terms *taste* and *flavor* are used interchangeably [8]. Extant literature considers taste as a constituent of the five senses and it has been proven that humans can detect only five taste qualities, which are sweetness, saltiness, sourness, bitterness and umami; on the other hand, the multisensory perception arising from foods in the oral cavity implies flavor [8]. Flavor is defined as “the sensation arising from the integration or interplay of signals produced as a consequence of sensing smell, taste and irritating stimuli from a food or beverage” [9] (p. 387). The perception of food taste and flavor is thus a multisensory experience, and it is considered ambitious to think that customers can distinguish between subtle tastes by only relying on our “taste” senses [10], as when it comes to eating and drinking, all five senses, including smell, touch, vision and audition, participate in enriching the consumption experience. For instance, research studies about flavor perception have pointed out that odors can cause changes in the perceived sweetness of food [11]. Regarding perceived healthiness, several studies examined the relationship between the construct and taste, showing the existence of a common belief among people that a food product cannot be healthy without sacrificing taste [12].

Despite the ongoing shift towards a healthier diet, many consumers still overconsume energy-dense foods either because consumers are unaware of the negative consequences of consuming such foods [3], or because unhealthy foods are usually associated or cued with attributes arousing flavor perception, which in turn drives food decisions [7].

Indeed, intrinsic and extrinsic cues are determinant factors analyzed by consumers—both passively or actively—able to affect the overall perception and quality of products [8,13,14,15,16]. Sensorial cues play a fundamental role in evaluating food and drinks [17], involving both human-side factors, such as genetic backgrounds (e.g., taste receptors) or physiological state (e.g., hunger), and food-related factors, such as contexts (e.g., eating environment), their appearance or extrinsic cues [8]. Each cue is relevant when developing impressions of the product itself [13]. An intrinsic cue refers to the attributes and physical properties inherent to the product itself (e.g., the type of grape used to produce a specific wine), such as color or fat content; these characteristics cannot be changed without changing the physical properties of the product [13]. An extrinsic cue, in contrast, is a piece of information about a product that comes from outside the product itself, such as price or packaging [8,14,15,16]. One of the typical questions is how can extrinsic cues influence taste and flavor experiences if they do not come directly from foods and beverages? As an explanation, some scholars consider “expectation” as a valuable mediator for the relationship between extrinsic cues and taste/flavor experiences. Expectations can be defined as “subjective notions of thing to come” or “pre-trial beliefs about the product” [8] (p. 249). Those expectations usually exist before experiencing taste and flavor, and during their formation, extrinsic cues, if available, are evaluated and elaborated through knowledge and memories related to the cues themselves [8]. Even though research shows that intrinsic cues play an important role when consumers form their opinions, in some situations, extrinsic cues are believed as more credible and reliable, as more influential than sensory perceptions [15].

There is plentiful evidence, indeed, that flavor experiences are affected by external influences, such as brand names, brand familiarity, store name, prices, country of origin, descriptions of taste/flavors, product information (e.g., food ingredients, nutritional information), product packaging, advertising and other “sensory-oriented” features, like physical attributes, the shape of symbols or the sound of names [8,10,18]. Some authors have also analyzed how the colors of packaging, environment and tableware can impact food expectations and perception [19,20]. Elder and Krishna (2010) [21] demonstrate that taste is also subject to extrinsic cues, such as the content of advertising, by indicating that multiple-sense advertising (vs. single-sense ads) increased taste perceptions.

Additionally, the information provided in the form of a label significantly affects flavor perception by creating a set of expectations about the product [22,23,24,25,26,27,28,29]. Other authors demonstrated the relevance of textual claims—such as preparation process, nutritional information—in affecting consumers’ perception of food [16,23,30,31]. Textual claims have different effects for specific product categories and specific consumer groups [32]. For instance, hedonic labels (as opposed to health labels) had a positive effect on consumer responses toward a hedonic product [33]. The effect was the opposite for a healthy product, where the health label increased product evaluation and purchase intention. In the setting of a real product purchase, packaging attributes play an important role as an extrinsic cue, providing information outside the product itself, able to influence consumers’ attitude and behavioral intention, as well as expectations on products’ healthiness and tastiness [8,14,15,16,34].

Emerging cues, which could impact consumers’ food evaluations and not yet in-depth investigated in the literature, are those supported or provided by technology, and specifically connected to blockchain. It is a technology with great potential, given its role in addressing trust issues in food supply chains in an easily accessible format, such as mobile devices for consumers [35,36]. One of the main reasons behind the adoption of blockchain technology is being nurtured, especially in developed countries such as Canada, which is its relation to food safety issues [37]. In Canada, indeed, “1 in 8 people (4 million Canadians) get sick each year from contaminated food” [38], and thus they expect that food purchased through a grocery retailer is safe to eat and will not cause health issues [39]. Blockchain plays a fundamental role in fighting counterfeit products, most of all pharmaceuticals, agricultural chemicals, luxury goods and electronics [40]. Firms of the agricultural industry are redefining and upgrading the strategies and approaches through which they interact with customers, providing them with more transparent information about the food provenance, production and ethical principles behind the value chain. Prior findings show that consumers are inclined to seek specific details on daily foods, including original materials and ingredients, production packaging, allocation, pesticide and hormone residue labels. In this sense, food traceability could be an effective solution to enhance consumer confidence and consumption through the communication of the safety characteristics of the food [41]. Some studies have discovered that traceability is highly rated by consumers for numerous reasons, such as the fact that it provides information about the origin of the animal and the location where the transformation process has taken place [42].

The different players in the agri-food value-chain, from the producer of the raw material to the packer/produced of end products, to the retailer/distributor are responding to this emerging need in different ways. Among others, one of the attempts is to implement blockchain technology, to supply detailed information about products, sources of materials, and production processes [43], with the double advantage of: (i) advancing the agri-food industry towards healthier and safer practices, and (ii) making consumers more informed [44,45], in this case about what they are eating, while nudging them towards a more balanced diet. An additional advantage is that multiple information might be made available in a friendly and accessible way to consumers, through QR codes and link texts showed on food packages [46,47]. Thus, consumers can easily access information in the retail shop, for example through interactive kiosks, and check it in every moment without consulting the internet. In addition, those messages are certified and “unchangeable”, thus implicitly increasing trust in the potential claim that appears on the packages, while providing transparency on the related supporting evidence. Previous research studies have shown that the use of QR codes by consumers could be beneficial, especially in the case of more expensive products, products with higher risk (e.g., medicines or exotic and healthy foods like seaweeds) [40]. Another element which helps reassure consumers about the truthfulness of the information provided is that certification agencies and regulatory bodies also participate in the supply chain system. In this case, their role consists in verifying the claims made by farmers, food processors or manufacturers regarding the product, such as their organic certificate, the chemicals used and the welfare status of the farm [48]. Furthermore, a blockchain-based solution provides consumer protection: this technology “creates digital records of consumer’s purchases, moving product warranties from paper onto the cloud via blockchain—keeping them up-to-date and easily transferable” [40].

In light of the above evidence, the scope of our research is to combine the informational cues provided by blockchain technology and applied on food packaging with consumer behavior, measured in terms of perceived flavor and perceived healthiness, addressing the following research questions:

RQ1. Does blockchain-certified information available on the package affect consumers’ perception of healthiness and flavor?

RQ2. Does the effect of blockchain-certified information vary among countries or animal milk categories?

With the aim to define whether blockchain-certified information is able to nudge consumers’ perceptions in food shopping contexts, we formulated the following research hypothesis to test:

**Hypothesis** **1** **(H1).** *The presence (vs. absence) of the blockchain technology on product packaging evokes effects on consumers’ food evaluation.*

Specifically:

**Hypothesis** **1a** **(H1a).** *Products cued with information certified with blockchain technology are perceived as tastier than products without blockchain technology on product packaging.*

**Hypothesis** **1b** **(H1b).** *Products cued with information certified with blockchain technology are perceived as healthier than products without blockchain technology on product packaging.*

**Hypothesis** **2** **(H2).** *The effect of information certified with blockchain technology on perceived healthiness varies according to the country.*

**Hypothesis** **3** **(H3).** *The effect of information certified with blockchain technology on perceived flavor varies according to the milk category*.

The remaining part of the paper describes the methodological approach, analyzes the results, discusses the results and introduces potential future research avenues.

## 2. Methods and Materials

The present study aims to answer the above research questions and hypotheses with a sequence of statistical analyses, structured through a between-subject experimental design to assess the potential mean differences across groups. We carried out a sequence of two analysis: (a) an *Analysis of Covariance* (ANCOVA) to test the effect of manipulation toward perceived healthiness and flavor while controlling for country and milk category to test H1; and (b) a two-way ANOVA to evaluate the mean differences according to the country and the category of milk frequently consumed to test H2 and H3.

### 2.1. Research Design and Stimuli

A between-subject design was used for this study, with two different conditions as stimuli: (1) it shows an empty package, without any recall to blockchain and certified information (control condition), and (2), which displays a QR Code, through which consumers access the aforementioned website and can also see an enlarged description of the elements, in order to clearly understand the information provided on the diet of the cow, its country of origin and health state, milk composition and drugs administered (intervention).

**Figure 1:**
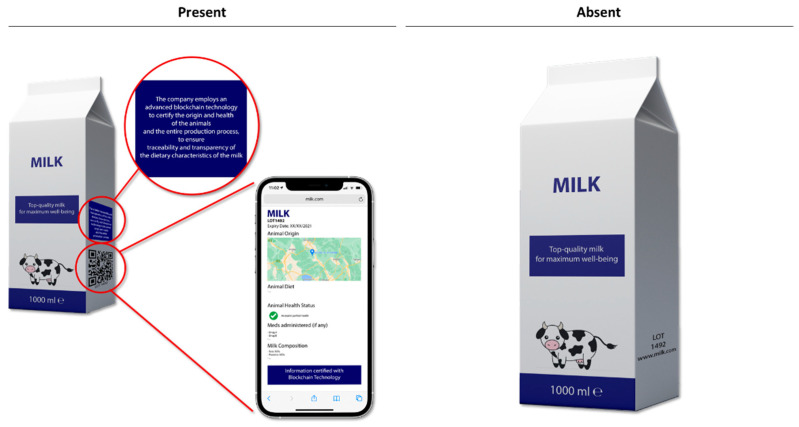
Mock-up product and conditions. Example UK version.

We included mock products to avoid additional influences exerted by the presence of a brand [49,50]. As shown in Figure 1, the packages resemble real milk packages under 2 alternative conditions, having in common the same benefit of “top quality milk for maximum well-being”.

The decision to include mock products is based on the idea of preventing branded additional information influencing participants’ perceptions of the products, as informed by similar research, e.g., [51,52,53,54,55]. The mock packages were realized to resemble real milk packages available in the grocery. To enhance our measurement validity and avoid errors derived from consumers’ perception of milk composition and product perception (e.g., different components and types of milk might impact on product price, like butterfat as the most expensive one and skimmed milk as being the cheapest [56]), we decided to create a mock-up aimed at providing only the necessary information for the test, without a priming on any sub-category. Products were provided without price as this might bias participants’ responses [57].

This study was carried out in three countries—the UK, Germany, and Italy. In each country, respondents were exposed to one randomized condition only. This resulted in a 2 (control vs. intervention) × 3 (tested countries), with a total of 6 different scenarios. To the extent of our knowledge, considering the novelty of blockchain technology in certifying supply chain and as a cue of the product, no other study has tested the effect of certified information available on a package on perceived flavor and healthiness in the aforementioned countries. We also chose such countries in accordance with their relevant role in milk production. In line with the statistics outlined by Eurostat (2019) [58], Italy and Germany are among the main milk producers in Europe, covering 31.1% of the entire production. Additionally, UK consumers frequently use milk in their diet, consuming 14,964 million L in milk production in the last year and 8144 L per cow per annum [59].

According to industry expert interviews, we utilized three different typologies of animal milk category: *skimmed milk, low-fat milk and whole milk.*

With the aim to contribute to the extant literature, this research outlines evidence on the effect of such cues in terms of perceived flavor and healthiness and whether differences exist among the three aforementioned countries and the different types of animal milk.

### 2.2. Study Population and Data Collection

A total of 360 individuals from the three countries, namely the United Kingdom (UK), Germany (GER), and Italy (ITA), were recruited through Prolific, a recently established international web panel provider, which combines high recruitment standards and the proper response rate, reliability and high replicability of studies [60]. Respondents were filtered according to their role in the purchase. Primary grocery shoppers were included regardless of their traditional gender roles within the household—highlighted by several authors [61,62,63,64]. According to the recent paradigm shift in purchases, the presence of both male, female and non-binary shoppers are on the rise, irrespective of traditional perspectives [64,65]. Furthermore, the filter ensured higher confidence that the interactions were associated with purchase-related tasks [66] and with a common product such as milk. Those who were not responsible for purchases were withdrawn from the study. After their agreement, participants were asked to complete an online survey, removing submissions with a response time lower than 3 min. This resulted in 310 individuals. The survey was submitted in the country’s official language. After having provided information concerning the role in purchases, respondents were randomly assigned to one of the two different conditions presented above; each respondent only saw one of the two stimuli. Subsequently, respondents were asked to answer questions aimed at measuring their perceived healthiness and flavor. Details of the sample size by country and socio-demographic information are provided in Table 1.

### 2.3. Constructs and Measures

The dimension of *perceived flavor* was measured using a set of items previously tested by Schouteten et al. (2015) [67]. We also added a specific set of measures for perceived healthiness adopting items tested by Fenko et al. (2016) [66]. In line with the items tested in past research studies, we utilized a set of measures derived from the extant literature, and also pre-validated in terms of their reliability for this study. Specifically:

*Perceived healthiness*, through 7-point Likert scale (1 = strongly disagree, 7 = strongly agree): “I expect this product to be healthy”, “I would consider this product as good for me”, “The product sounds healthy”, “The product sounds healthy”, “The product looks healthy”, “This product looks low in calories”, “I have an impression that this product is healthy”, “This product looks healthier than similar category products” (α = 0.902);

*Perceived flavor* items, through a 7-point Likert scale (1 = not at all, 7 = extremely): “How much do you think you will like this milk”, “How savory do you think this milk will taste?”, “How flavorful do you think this milk will taste?”, “How delicious do you think this milk will taste?”,” How much do you want to taste this milk?” (α = 0.876).

### 2.4. Statistical Analysis

The analyses were carried out using SPSS Statistics (version 25, SPSS Inc., Chicago, IL, USA). After evaluating the reliability of measured scales, we tested means for perceived healthiness and flavor controlling for *country* and *milk category* and then assessed mean differences according to the analyzed country and the milk category frequently consumed. An *analysis of covariance* (ANCOVA) was conducted to test whether the means of each dependent variable (i.e., healthiness and flavor) were equal across the levels of the categorical independent variables (manipulation variable), while statistically controlling for the effects of country and milk category. In a subsequent two-way ANOVA, interactions between condition and country, and condition and milk category were included as independent variables.

A post hoc test was performed to deeply assess the mean differences of each stimulus across countries. The estimated marginal means for different stimuli and the stimuli by country and milk category interactions were plotted for all the dependent variables. We included the milk category as the control variable in accordance with the findings deriving from interviews with expert milk producers. They posited that consumers activate different buying responses according to the consumed category. In line with this perspective, this study expanded the evidence of interviews through a survey administered to a larger sample of European consumers. Additionally, to assess whether different a cultural background affects the expectations formed after the exposure to an extrinsic cue (CIT.), we controlled for the country of respondents.

## 3. Results

Therefore, we outlined our results (1) combining the effect of manipulation cued with blockchain-certified information on healthiness and flavor; and (2) highlighting the main differences for each country and milk category.

### 3.1. Main Effects of Blockchain-Certified Information on Perceived Flavor and Healthiness

Through an ANCOVA, we validated our results assuming both healthiness and perceived flavor as dependent variables while controlling for *country* and *milk category*. As showcased in Table 2, perceived flavor significantly varied across the two levels of our manipulation variable (*F(1, 306)* = 9.025, *p* = 0.003), reporting a different mean according to the control (*M_Control_* = 4.659; *SD* = 0.081) and the intervention group (*M_Intervention_* = 4.998; *SD* = 0.078). Thus, the H1a is not rejected. Such evidence suggests that individuals exposed to certified information with blockchain (e.g., about the health, location, and nutrition of cows) tend to form different evaluations about the flavor perception of animal milk when compared to the control group. Furthermore, we found that the *milk category* (i.e., *skimmed milk, low-fat milk, and whole milk*) was a significant covariate affecting the perceived flavor (*F(1, 306)* = 10.281, *p* = 0.001), suggesting further investigations in assessing the differences according to each category. Contrarily, the country was not a relevant covariate (*F(1, 306)* = 0,915, *p* = 0.340), indicating there are no significant mean differences in the effect of the manipulation on flavor according to the analyzed country.

**Figure 2:**
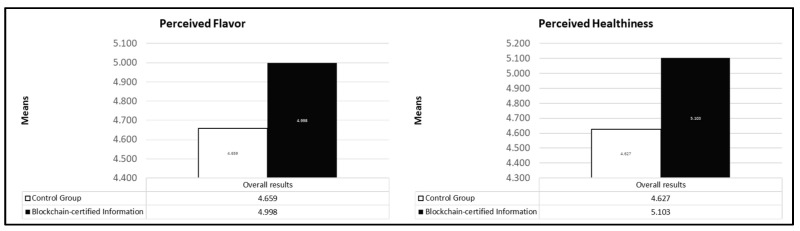
Adjusted mean by condition. Both differences are significant (*p* < 0.05).

In the same vein, we repeated the analysis with healthiness as the dependent variable, also assuming *country* and the *milk category* as covariates. Overall, the model was significant (*F(3, 306)* = 7.845, *p* = 0.000), allowing us to proceed analyzing the main effects of each variable. According to the results, a significant mean difference was found between the control and the intervention group, where blockchain-certified information was highlighted (*F(1, 306)* = 16.056, *p* = 0.000). However, planned contrasts showed a significant mean difference of *MD* = 0.476 between the control group (*M_Control_* = 4.627; *SD* = 0.085) and the group exposed to certified information (*M_Intervention_* = 5.103; *SD* = 0.082) (Figure 2). Hence, H1b is not rejected. This means that individuals, when exposed to a sequence of blockchain-certified information (e.g., regarding the nutrition, country of origin, and drugs administered to the cow), perceive the product as healthier when compared to the absence of these data. As in the previous case, we found a significant effect of *milk category* on healthiness (*F(1, 306)* = 5.418, *p* = 0.021) suggesting potential differences according to the category consumed by individuals. However, the country is not a significant covariate (*F(1, 306)* = 0.418, *p* = 0.000). Such results showcased a positive effect of blockchain technology, used as a certification protocol for product attributes and processing, on perceived flavor and healthiness. Furthermore, outputs suggested that significant covariates, as the milk category, can be further assessed.

### 3.2. Assessing Mean Differences by Milk Category and Country

A two-way ANOVA was carried out to test whether significant effects of blockchain-certified information on healthiness and flavor varied according to the *milk category* or *country.* We tested the effect of manipulation, *milk category,* and their interaction on flavor and healthiness. We then carried out the analysis substituting *country* for *milk category*.

In line with previous results, a two-way ANOVA showed significant mean differences of blockchain-certified information on healthiness according to the category consumed. However, as in the previous case, manipulation (*F(1, 304)* = 13.635; *p* = 0.000) and *milk category* (*F(2, 304)* = 3.080; *p* = 0.047) consistently affects healthiness whilst their interaction is not significant (*F(2, 304)* = 1.861; *p* = 0.000). Similar results have been outlined considering flavor as a dependent variable. The manipulation (*F(1, 304)* = 7.841; *p* = 0.005) and *milk category* (*F(2, 304)* = 5.616; *p* = 0.004) significantly affects the perceived flavor while their interaction has not been found to be significant (*F(2, 304)* = 0.824; *p* = 0.440). Furthermore, results suggest that the effect of blockchain-certified information is irrespective of the observed country. The two-way ANOVA demonstrated that flavor perception does not vary significantly b Italy, the UK and Germany (*F(2, 304)* = 0.663; *p* = 0.516). Comparably, the perceived healthiness does not significantly vary according to the country (*F(2, 304)* = 1.711; *p* = 0.438).

**Figure 3:**
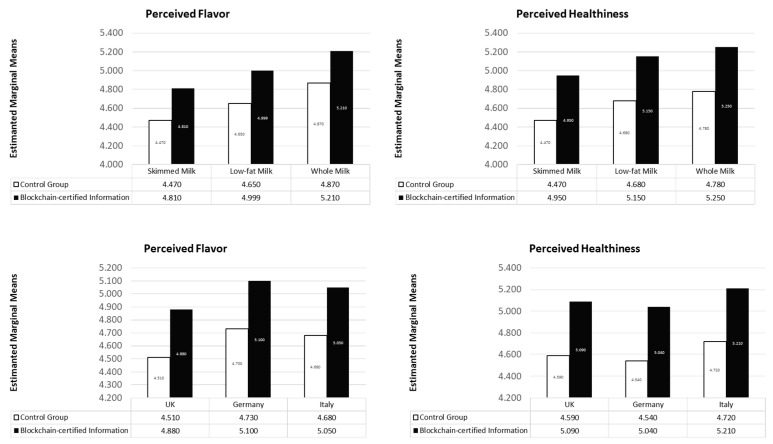
Mean differences by milk category and country. Only skimmed milk vs. whole milk difference is significant (*p* < 0.05).

We deepened the aforementioned results through a post hoc test evidencing the significant mean differences existing according to the subgroups of *milk category.* However, we found that a significant mean difference of *MD* = −0.4402 (*p* = 0.001) occurs in the combination of skimmed milk vs. whole milk, describing different flavor perceptions based on the milk consumed. However, whole milk consumers tend to score higher evaluations on product flavor when compared to skimmed milk consumers. Comparably, individuals who consume skimmed milk tend to perceive the product as less healthy when compared to whole milk consumers (*MD* = −0.354; *p* = 0.021). Such evidence could be related to the presence of fat information on the package. These cues could affect the evaluation of the healthiness while evidencing the real milk composition. Other pairwise combinations were not found to be significant (Figure 3).

## 4. Discussion

Blockchain technology is increasingly supported by a sequence of regulatory initiatives and incentives to find an application in agri-food contexts and to shed light on supply chain processes, delivering to consumers a reliable set of information about the product, such as the Demeter Horizon 2020 project [68]. Food information certified with blockchain could also appear on shelves in the years to come, thus providing a link to product’s details through a QR code or labeling system. This would result in a new extrinsic cue which could affect consumers’ expectations about the healthiness and flavor, as others have previously tested [22,23,24,25,26,27,28,29]. Prior studies conducted in 2019 reported that blockchain technology was the most used technology in the field of food tracking in Italy, being utilized by 43% of the whole supply chain, while 41% of them provided information through QR codes; moreover, food products were tracked with mobile applications in 36% of cases [69].

The present study focused on blockchain-certified information presented on a package of animal milk, assessing the effects of such cues on its perceived flavor and healthiness.

The current study highlights that blockchain-certified information can be considered an effective additional external cue, as it regards the perceived flavor and healthiness, adding to the existing literature which previously assessed the role of other external factors, such as brand, country of origin or food ingredients and nutritional information in influencing consumers’ pre-trial beliefs about the product [8]. The intensity of the results varies in accordance with the category of milk consumed by individuals. Combining all milk category consumers, products cued with certified information are perceived as more flavored when compared to control groups. In contrast, deepening existing differences, whole milk consumers significantly perceive certified products as having more flavor than skimmed milk consumers do. Such results deserve further investigation with the aim to explore whether specific information moderates the perceived flavor of the product. As previously mentioned, increasing the information provided to consumers about the product could shed light on some aspects that could negatively influence consumers. Country of origin, place of breeding, drugs administered to the cow or milk composition could affect the expectations of consumers and subsequently their perceived flavor.

The results also suggested the positive effect of certified information in the consumer perception of food healthiness. Such evidence could be explained by considering the role of this technology in providing an enlarged set of product information, involving the nutrition of the cow, its country of origin, health state, or drugs administered. These details are mainly focused on the habitat and status of the animal, and their appearance reduces the credence sphere of the product while providing real information about the lifecycle. Otherwise, consumers of skimmed milk are less inclined to perceive the product cued with certified information as healthier. It might derive from the fat level reported in the stimulus. However, by increasing the information provided on the product, consumers could vary their perceptions due to the fat level or other attributes attached to the product itself. Moreover, information value increases when generated from different and independent sources. In fact, health-conscious consumers might use such information to avoid the consumption of the product focusing on milk composition (i.e., fat level) while health-indifferent consumers increase their flavor perception by reading details about the story, the habitat and the status of the animal.

Accordingly, in recent years, scholars have identified relevant differences, which are (i) the course of human development (e.g., adulthood vs. childhood); (ii) a person’s taster status (e.g., supertasters vs. medium tasters vs. non-tasters); (iii) cross-cultural differences in the meaning of colors; (iv) the degree of a person’s expertise that would affect consumers’ perceptions and attitude of a product [70,71]. Considering that companies, institutions, and society at large are implementing actions to reformulate the nutritional information presented on food packages [72], also supporting the introduction of other forms of certifications aimed at providing details on supply chains and products lifecycle, the investigation of such influent products’ cues could shed some light on real factors affecting decisions and in turn consumers’ habit and diets.

These initiatives represent an important opportunity, for both scholars and firms, to examine which products’ cues could arouse the perception of healthier and flavor product, contrasting the consumption of products with high levels of sugars, sodium, and saturated fats, also evaluating the interplay of those cues with front-of-pack labels [73,74] in light of the new upcoming “from farm to fork” strategy.

However, some limitations of the study should be mentioned. First, future research could engage a higher number of respondents [75] by enlarging the sample size, thus increasing the external validity of results and extending the quota sample to further maximize representativeness in Italy, UK and Germany. Second, as previously mentioned, mock products have been shown without price as it might bias participants’ responses [57]. Given the importance of price in food purchases [76], the role of pricing could be explored in future contributions. In addition, the fact that the information displayed on the packages, such as fat content, brand name and colors, can affect purchasers’ judgments, creating expectations about the foods, also in terms of perceived healthiness and flavor [22,23,24,25,26,27,28,29], which could be taken into consideration. Similarly, implementing blockchain technology means providing detailed information about products, sources of materials and production processes [43]. Nevertheless, some scholars have demonstrated that consumers spend on average between 23 and 29 s purchasing products or they act routinely [77], providing a great amount of information, such as those supplied by blockchain technology, which could also have negative consequences. Moreover, according to the Information Overload Theory, “consumers are limited in terms of the amount of information they can assimilate and process at a certain time” and exceeding these limits will cause an overload [78] (p. 30). It could be then explored whether any overload of information is present and whether this can complicate the decision-making process. Additionally, information certified with blockchain technology could be supplied by both independent and internationally accepted certification bodies, which can decrease consumers’ trust. There remains a lack of evidence about the impact of blockchain on the consumer goods sector [79]. Its expensiveness and redundancy may far outweigh the values derived from its applications for marketing [80].

Moreover, the ownership structure of firms is a relevant factor taken into account by consumers when making purchasing decisions [81], while others may pay premium prices for the humane treatment of animals [82]; thus, future studies might explore how consumers value the type of ownership of a farm, e.g., family-owned or a worker-owned cooperative farm, when considering milk purchases and also investigate the impact that fair and humane animal treatments can have on consumers. Another interesting element concerns the potential role that blockchain-enabled loyalty programs could have in brand storytelling related to flavor and healthiness. Blockchain technology is constantly increasing its popularity, and through some blockchain-based platforms, it is possible to order branded goods directly from the manufacturer; related to this, consumers may be able to pay in different ways, such as FIAT currency or cryptocurrencies (e.g., Bitcoin) [40]. In our study, we did not concentrate on the usage of the milk purchased by consumers, e.g., whether they intend to drink it or to use it in cooking; therefore, future studies may deepen this topic and explore how and whether the perceived tastiness varies according to milk usage. Finally, further study might also concentrate on the point of view of the producers, and their perception of how transparency provided by blockchain might affect the way they conduct their business [83].

## 5. Conclusions

Blockchain technology-certified information, when cued on products, affects consumers’ perceptions of flavor and healthiness. Such results vary according to the category of milk consumed irrespective of the country of origin. Further studies might be opened to deep dive into the study of the mediating and moderating effects of tech-mediated information on food perception and understand the combination of cues and factors which could stimulate the consumption of specific products.

## Figures and Tables

**Table 1:** Details of sample size by country and socio-demographic information.

Variables	Italy	UK	Germany
	*n* = 102	*n* = 108	*n* = 100
Age			
18–24	10.8%	12.0%	5.6%
25–34	18.6%	10.0%	42.6%
35–44	31.4%	24.0%	25.0%
45–54	17.6%	25.0%	15.7%
55–64	19.6%	20.0%	10.2%
65+	2.0%	9.0%	0.9%
Education			
Lower than diploma	0.0%	23.0%	13.9%
Diploma	27.5%	22.0%	16.7%
Bachelor’s degree or equivalent	22.5%	42.0%	30.6%
Master’s degree or equivalent	38.2%	11.0%	30.5%
Ph.D.	11.8%	2.0%	8.3%
Occupation			
Full-time employee	53.0%	45.0%	50.9%
Part-time employee	23.0%	28.0%	28.7%
Unemployed, seeking a job	9.0%	6.0%	6.5%
Unemployed, not seeking a job	3.0%	7.0%	3.7%
Retired	3.0%	12.0%	1.9%
Student	9.0%	2.0%	8.3%
Income			
Less than EUR 10,000	18.6%	16.0%	20.4%
EUR 10,000–EUR 19,999	20.6%	26.0%	25.0%
EUR 20,000–EUR 39,999	41.2%	39.0%	26.0%
EUR 40,000–EUR 59,999	7.8%	15.0%	9.0%
EUR 60,000–EUR 79,999	9.8%	2.0%	11.1%
EUR 80,000–EUR 99,999	2.0%	1.0%	5.7%
More than EUR 100,000	0.0%	1.0%	2.8%

**Table 2:** Results of the analysis of covariance (ANCOVA).

Predictor	Perceived Flavor						Perceived Healthiness					
	SS	df	MS	*F*	*p*	*η^2^*	SS	df	MS	*F*	*p*	*η^2^*
Manipulation (A)	21.427	1	8.753	9.025	**	0.029	17.321	1	17.321	16.065	***	0.05
Country (B)	0.888	1	0.888	0.915		0.003	0.451	1	0.451	0.418		0.001
Milk Category (C)	9.971	1	9.971	10.281	**	0.033	5.845	1	5.845	5.418	*	0.017
Error	296.77	306	0.97				330.12	306				

(i) Perceived flavor: R^2^ = 0.067; perceived healthiness: R^2^ = 0.071. (ii) SS, sum of squares; df, degrees of freedom; MS, mean square; *F*, *F*-test; *η*^2^, effect size; *** *p* < 0.001; ** *p* < 0.01; * *p* < 0.05. (iii) Country and milk category are covariates.
